# Increased Expression of α-Hemoglobin Stabilizing Protein (AHSP) mRNA in Erythroid Precursor Cells Isolated from β-Thalassemia Patients Treated with Sirolimus (Rapamycin)

**DOI:** 10.3390/jcm13092479

**Published:** 2024-04-24

**Authors:** Matteo Zurlo, Cristina Zuccato, Lucia Carmela Cosenza, Maria Rita Gamberini, Alessia Finotti, Roberto Gambari

**Affiliations:** 1 Department of Life Sciences and Biotechnology, Ferrara University, 44121 Ferrara, Italy; matteo.zurlo@unife.it (M.Z.); cristina.zuccato@unife.it (C.Z.); luciacarmela.cosenza@unife.it (L.C.C.); 2 Center “Chiara Gemmo and Elio Zago” for the Research on Thalassemia, Department of Life Sciences and Biotechnology, Ferrara University, 44121 Ferrara, Italy; gamberinimariarita@gmail.com

**Keywords:** alpha-hemoglobin stabilizing protein, β-thalassemia, sirolimus, rapamycin, erythroid precursor cells (ErPCs)

## Abstract

**Background/Objectives**: in β-thalassemia, important clinical complications are caused by the presence of free α-globin chains in the erythroid cells of β-thalassemia patients. These free α-globin chains are present in excess as a result of the lack of β-globin chains to bind with; they tend to aggregate and precipitate, causing deleterious effects and overall cytotoxicity, maturation arrest of the erythroid cells and, ultimately, ineffective erythropoiesis. The chaperone protein α-hemoglobin-stabilizing protein (AHSP) reversibly binds with free α-globin; the resulting AHSP-αHb complex prevents aggregation and precipitation. Sirolimus (rapamycin) has been previously demonstrated to induce expression of fetal hemoglobin and decrease the excess of free α-globin chain in the erythroid cells of β-thalassemia patients. The objective of this study was to verify whether sirolimus is also able to upregulate AHSP expression in erythroid precursor cells (ErPCs) isolated from β-thalassemia patients. **Methods:** the expression of AHSP genes was analyzed by measuring the AHSP mRNA content by real-time quantitative PCR (RT-qPCR) and the AHSP protein production by Western blotting. **Results:** AHSP gene expression was found to be higher in ErPCs of β-thalassemia patients in comparison to ErPCs isolated from healthy subjects. In addition, AHSP expression was further induced by treatment of β-thalassemia ErPCs with sirolimus. Finally, AHSP mRNA was expressed at an increased level in ErPCs of sirolimus-treated β-thalassemia patients participating in the NCT03877809 Sirthalaclin clinical trial. **Conclusions:** this exploratory study suggests that AHSP expression should be considered as an endpoint in clinical trials based on sirolimus.

## 1. Introduction

The clinical severity of the hereditary β-thalassemia syndromes is primarily caused by mutations of the β-globin gene [1,2]. More than 350 mutations causing β-thalassemia have been reported so far [3,4]; however, only 20 mutations account for more than 80% of the β-thalassemia mutation worldwide [4]. These mutations cause a large variety of phenotype alterations, leading to low or absent production of adult hemoglobin [1,2]. For this reason, reactivation of γ-globin gene expression and increased fetal hemoglobin (HbF) levels have been proven beneficial to β-thalassemia patients [5,6]. A second and very important clinical complication is the presence of an excess of free α-globin chains in β-thalassemia erythroid cells due to the lack of β-globin chains to bind with [7,8]. These α-globin chains tend to bind with each other and precipitate, causing deleterious effects and overall cytotoxicity to erythroid cells. In addition, maturation arrest and apoptosis of erythroid precursors, reduction in the lifespan of circulating red blood cells, hemolysis and ineffective erythropoiesis have been reported [9,10,11,12].

In populations characterized by a high frequency of thalassemia, the co-inheritance of α- and β-thalassemia is not rare [1]. The hematological and clinical phenotypes of these complex genotypes help in understanding the deleterious effects of the excess free α-globin chains. In fact, diminished levels of excess α-globin chains were found in β-thalassemia heterozygotes with co-inheritance of α- and β-thalassemia. This is usually associated with a less severe clinical phenotype [1,8].

In β-thalassemia, several pathways operate to prevent and/or counteract the excess of free α-globin chains. Understanding these pathways is a key factor for designing novel pharmacological approaches to reduce ineffective erythropoiesis.

First of all, it is clearly established that protein quality control (PQC) pathways, such as ubiquitin-mediated α-globin proteolysis, are activated in β-thalassemia [13,14,15]. In fact, the portion of misfolded free α-globin is efficiently polyubiquitinated and degraded by the proteasome machinery [14,15]. This α-globin–specific proteolysis is increased in β-thalassemic erythroblasts relative to normal patient samples [16,17]. Interestingly, Khandros et al., using the heterozygous thalassemia (Th3/+) mouse model system, found an accumulation of insoluble α-globin chains in erythroid precursors. This insoluble α-globin chains fraction further accumulated in reticulocytes from (Th3/+) thalassemic mice in which the proteasome was inhibited [18].

In addition, a complementary biological process leading to reduced excess of free α-globin in erythroid cells is the autophagy process, dependent on the Unc-51-like autophagy activating kinase 1 (ULK1) [19,20,21]. In this context, Lechauve et al. published a key study demonstrating that an ULK1-dependent autophagy process was able to reduce ineffective erythropoiesis by decreasing the free α-globin. This process was found to be stimulated in a mouse model by the mTOR inhibitor rapamycin, demonstrating that the ULK1-dependent autophagy pathway is a druggable target [19]. The reduction of ineffective erythropoiesis was associated with an improvement in the life span of β-thalassemia red-blood cells [19].

A further biological process counteracting the deleterious effects of the excess of free α-globin in erythroid cells of β-thalassemia patients is based on the expression of the chaperone protein alpha-hemoglobin-stabilizing protein (AHSP) [22,23,24]. AHSP reversibly binds with free α-globin, generating an AHSP-αHb complex that prevents aggregation and precipitation of the free α-globin [25]. Therefore, we were interested in performing pilot experiments to verify whether induction or enhancement of the expression of the AHSP gene should be considered in clinical trials involving β-thalassemia patients [26,27,28,29,30,31,32].

The role of AHSP in β-thalassemia has been extensively studied [23,24,26,27,28,29,30,31,32,33,34] and also confirmed using animal model systems. For instance, Kong et al. generated *AHSP*^–/–^ mice by gene targeting [24]. As expected, abnormal erythrocyte morphology with hemoglobin precipitates was found in these animals [24]. Furthermore, they found that the loss of AHSP production reduced the lifespan of circulating RBCs and increased ROS-dependent apoptosis of erythroid precursors. Importantly, when informative interbreeding of mutant mice was performed, it was found that loss of AHSP production exacerbated the severity of β-thalassemia [24].

Sirolimus (rapamycin) is one of the most promising drugs for inducing HbF [35,36,37,38] and, at the same time, ULK1-mediated autophagy [19,20]. The several applications of sirolimus in biomedicine, from its discovery to its possible use in clinical trials, have been recently reviewed [39]. Sirolimus was first demonstrated to be a potent anti-bacterial and anti-fungal agent. Later, it was found to inhibit the in vitro cell growth of tumor cell lines. More importantly, for biomedical applications in the field of organ transplantation, sirolimus exhibits immunosuppressive activity [39]. More recently, sirolimus was found to be a potent inducer of fetal hemoglobin (HbF) in erythroid precursor cells isolated from normal subjects and β-thalassemia patients [35,36], in experimental in vivo mouse model systems [37,38] and in patients under sirolimus treatment [40,41]. Considering these activities, sirolimus was proposed as a repurposed drug of possible interest for clinical trials on β-thalassemia patients [39,42,43].

In this respect, sirolimus has been employed in pilot clinical trials on β-thalassemia patients (NCT03877809 and NCT04247750) [42]. The main objective of these two studies was to obtain biochemical and molecular evidence for verifying the possible efficacy of sirolimus as an in vivo inducer of HbF. While the results of the NCT04247750 trial have not been published yet, the RT-qPCR data obtained during the NCT03877809 trial [43] demonstrate an increase in γ-globin mRNA in peripheral blood and ErPCs of sirolimus-treated β-thalassemia patients. In addition, sirolimus was found to influence erythropoiesis, reducing ineffective erythropoiesis [43].

Interestingly, Zurlo et al. recently reported increased *ULK1* mRNA and decreased free α-globin content in erythroid precursor cells (ErPCs) isolated from β-thalassemia patients participating in the NCT03877809 clinical trial and treated with sirolimus [20]. This finding supports the concept that autophagy, ULK1 expression, and α-globin chain reduction should be considered important endpoints in sirolimus-based clinical trials for β-thalassemia [20].

The objective of the present study was to verify the hypothesis that rapamycin could have in vitro and in vivo effects on the expression of the *AHSP* gene in order to have a more complete understanding of sirolimus effects on β-thalassemia patients regarding ineffective erythropoiesis. In this respect, there is a lack of literature on this specific issue; in fact, the effects of the several HbF inducers described so far on *AHSP* gene expression have not been analyzed in depth. In our study, the effects on *AHSP* gene expression have been analyzed by RT-qPCR using RNA extracted from ErPCs treated in vitro with sirolimus or isolated from sirolimus-treated β-thalassemia patients participating in the NCT03877809 clinical trial.

## 2. Materials and Methods

### 2.1. Culture and Treatment of Human Erythroid Precursor Cells (ErPCs)

The employed two-phase liquid culture procedure has been described in detail elsewhere [35,44,45]. Briefly, mononuclear cells were isolated and cultured for 7 days [35]. After this phase I culture, non-adherent cells were cultured in phase II medium, in the presence of 1 U/mL human recombinant erythropoietin (EPO) (Tebu-bio, Magenta, Milano, Italy) and stem cell factor (SCF, BioSource International, Camarillo, CA, USA, 10 ng/mL). After 5 days of phase II culture, cells were treated with sirolimus (Sigma-Aldrich, St Louis, MO, USA) for an additional 5 days [35]. Using this experimental system, two approaches were considered to verify rapamycin’s effects on the expression of AHSP.

The first was an ex vivo approach. In this case, the ErPCs samples were isolated from three healthy subjects and a total of nine β-thalassemia patients with different genotypes (five patients were β^0^ 39/β^0^ 39, one patient was β^0^39/β^+^IVSI-110, two patients were β^+^IVSI-110/β^+^IVSI-110, one patient was β^+^IVSI-6/β^+^IVSI-6). For comparison, each cohort was selected in order to have study groups homogenous with respect to age (considering that the age of the patients was between 40 and 60 years). The ErPC populations were either untreated or treated with a low concentration of sirolimus (200 nM) as already described [35].

The second was an in vivo approach. In this case, the molecular and biochemical analyses have been conducted on ErPCs isolated from all the eight β-thalassemia patients participating in the Sirthalaclin NCT03877809 clinical trial and daily treated with 1 mg/day sirolimus for at least 90 days. Before analyses, the isolated ErPCs were cultured following the two-phase liquid culture procedures, performed in the presence of 1U/mL EPO in the absence of sirolimus. This experimental strategy, as well as the selection criteria for the recruitment of patients participating in the Sirthalaclin NCT03877809 clinical trial, have been described by Gamberini et al. [42] and by Zuccato et al. [43].

### 2.2. RT-qPCR Analysis

To measure changes in *AHSP* gene expression, the *AHSP* mRNA was quantified by Reverse Transcription-quantitative-Real Time PCR (RT-qPCR). The total cellular RNA was extracted using TRI Reagent® (Sigma-Aldrich, St Louis, MO, USA), as described elsewhere [43].

For gene expression analysis, as recently reported, 300 ng of total RNA was reverse transcribed using the PrimeScript RT kit from Takara Bio (Takara Bio Inc., Shiga, Japan) [35,43]. To quantify the expression of the *AHSP* gene, quantitative real-time PCR assay (RT-qPCR) was performed, and the *AHSP* mRNA content was compared to the house-keeping sequences *GAPDH*, *RPL13A*, and β*-actin* (probes and primers reported in Table 1).

Each reaction mixture contained 1× TaKaRa Ex Taq^®^ DNA Polymerase (Takara Bio Inc., Shiga, Japan). 300 nM PCR primers and 200 nM probes (Integrated DNA Technologies, Castenaso, Italy) were employed using CFX96 Touch Real-Time PCR System (Bio-Rad, Hercules, CA, USA). The following protocol was used: initial denaturation at 95 °C, 1 min; 50 PCR cycles performed (95 °C for 15 s, 60 °C for 60 s). The CFX manager software (Bio-Rad, Hercules, CA, USA) was employed for data analysis using the ΔΔCt method for quantification [43].

### 2.3. Western Blotting

In order to measure changes in AHSP protein content, AHSP was quantified in ErPC cytoplasmic extracts by Western Blotting. Before running the gels, protein concentration in these extracts was determined using PierceTM BCA Protein Assay Kit (Thermo Fisher, Waltham, MA, USA). Twenty μg of cytoplasmic extracts were denatured for 5 min at 98 °C in SDS sample buffer (Cell Signalling Technology, Danverss, MA, USA) and loaded on hand cast SDS-PAGE 14% gel (10 cm × 8 cm) in Tris-glycine Buffer (Bio-Rad, Hercules, CA, USA). Then, electro-transfer to 0.2 μm pore size nitrocellulose membrane (Thermo Fisher, Waltham, MA, USA) was performed overnight at 360 mA and 4 °C in standard Tris-Glycine-MeOH transfer buffer. After washing in TBS-T, the membranes were incubated with the primary antibodies. The different phases of the methods are fully described in Finotti et al. [46]. Bio-Rad Image Lab Software (Bio-Rad, Hercules, CA, USA) was used to analyze the blot images. The employed antibodies are reported in Table 2.

### 2.4. Statistical Analysis

All the data were normally distributed and presented as mean ± S.D. When appropriate, statistical differences between groups were compared using the Prism Software v9.02 and unpaired or paired t-tests. Statistical differences were considered significant when *p* < 0.05 (*), and highly significant when *p* < 0.01 (**), as reported elsewhere in similar studies [43,47].

## 3. Results

### 3.1. Production of AHSP in β-Thalassemic Erythroid Precursor Cells (ErPCs): Comparison with ErPCs from Healthy Subjects

**Figure 1:**
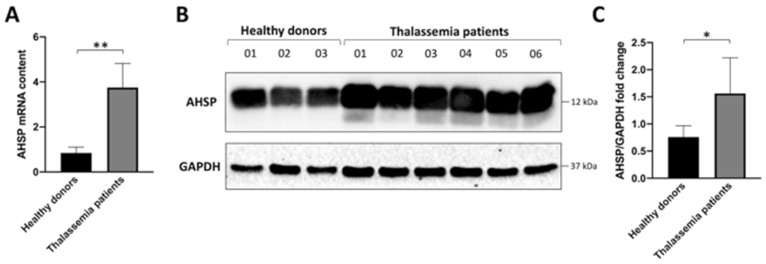
Expression of AHSP in Erythroid Precursor Cells (ErPCs) isolated from healthy subjects and β-thalassemia patients. (**A**). RT-qPCR analysis showing the relative content of *AHSP* mRNA (*GAPDH* was chosen as internal control). (**B**,**C**). Western blotting analysis. Autoradiograms are shown in panel (**B**) (the uncut version of the gels is shown in Supplementary Figure S2), the densitometric quantification of obtained bands is shown in panel (**C**). * = *p* < 0.05 (statistically significant); ** = *p* < 0.01 (statistically highly significant); unpaired *t*-test was employed.

In order to measure changes in *AHSP* gene expression, we first analyzed the content of *AHSP* mRNA by Reverse Transcription-quantitative-Real Time PCR (RT-qPCR). Figure 1A shows the relative content of *AHSP* mRNA in ErPCs isolated from healthy subjects and six β-thalassemia patients carrying different genotypes. Four patients were β^0^ 39/β^0^ 39, one patient was β^0^ 39/β^+^IVSI-110, and one patient was β^+^IVSI-110/β^+^IVSI-110. The results clearly show that *AHSP* mRNA is expressed at higher levels in ErPCs from β-thalassemia patients, after comparison with healthy subjects (Figure 1A). Similar results were obtained using different internal control sequences (RPL13A and β-actin), as depicted in Supplementary Figure S1. These data are in agreement with the Western blotting analysis shown in panel B of Figure 1, designed to quantify the content of the AHSP in ErPCs. As expected from previously reported studies [29,30,31], the content of AHSP was found to be high in ErPCs from both healthy and β-thalassemia patients. However, the content was higher in ErPCs from β-thalassemia patients, as also depicted in the quantitative analysis shown in Figure 1C. Considered together, these results suggest that the intracellular content of AHSP (both mRNA and protein) is higher in β-thalassemia patients with respect to healthy subjects.

### 3.2. Sirolimus (Rapamycin) Induces Increase in AHSP mRNA Expression in ErPCs from β-Thalassemia Patients

**Figure 2:**
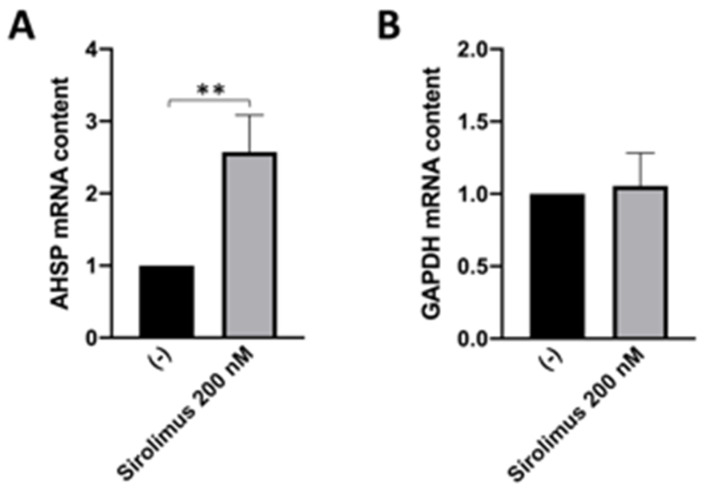
(**A**). Effect of sirolimus treatment on *AHSP* mRNA accumulation in ErPCs isolated from three β-thalassemia patients. (**B**). *GAPDH* gene expression normalized on β-actin, showing no difference following sirolimus treatment. The quantification of the *AHSP* mRNA was performed using β-actin as internal reference sequence, as elsewhere described. The genotypes of the patients were β^+^IVSI-6/β^+^IVSI-6, β^0^ 39/β^0^ 39, β^+^IVSI-110/β^+^IVSI-110. ** = *p* < 0.01 (statistically highly significant). Data and relative analyses were generated using the Prism Software v9.02 and paired *t*-test.

Figure 2A shows that ex vivo treatment of ErPCs from β-thalassemia patients with 200 nM rapamycin induces a statistically significant increase in the *AHSP* mRNA content. As reported in previously published studies [19,35,36,43], this treatment induces a sharp and highly reproducible increase in the expression of γ-globin genes. The response to sirolimus (i.e., an increase in production of HbF) of the ErPCs from these three β-thalassemia patients has already been reported [20]. On the contrary, the content of *GAPDH* mRNA was found unchanged following this treatment (Figure 2B).

A summary of the effects of sirolimus on ErPCs from β-thalassemia patients has been recently reported by Zuccato et a. [43]. These data strongly support the concept that sirolimus, in addition to the reported effects on HbF and γ-globin gene expression [35,36,39,43,47], is able to activate programs controlling the excess to *α*-globin production, ultimately reducing ineffective erythropoiesis. In order to verify whether the sirolimus-mediated effect on *AHSP* mRNA also occurs in vivo in sirolimus-treated β-thalassemia patients, we took advantage of the clinical trial NCT03877809 (Sirthalaclin). The protocol of this clinical trial concerning the inclusion and exclusion criteria can be found in the report published by Gamberini et al. [42].

### 3.3. Selected Activities of the Sirthalaclin NCT03877809 Clinical Trial

**Figure 3:**
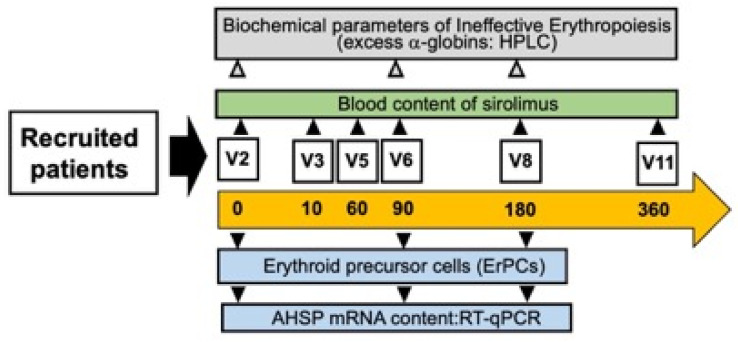
Scheme of this study, based on the Sirthalaclin NCT03877809 clinical trial. Modified from Zuccato et al. [43].

The first set of results obtained in the Sirthalaclin NCT03877809 clinical trial (Figure 3) have been reported by Zuccato et al. [43]. Additional information can be found in two studies reported by Zurlo et al. [20,47]. Relevant to the present study, these are the key points: (a) the sirolimus blood concentrations ranged from 1.0 ng/mL to 4.6 ng/mL, confirming that, under these conditions, the blood concentrations of sirolimus were low compared to other sirolimus-based clinical trials [48]; (b) despite these low concentrations in sirolimus-treated β-thalassemia patients, the expression of γ-globin mRNA increased in blood and ErPCs [26]. Sirolimus also reduced biochemical markers associated with ineffective erythropoiesis (such as excess of free α-globin chains). Interestingly, a decrease in the transfusion demand was observed in sirolimus-treated patients [43]. No alteration of the lymphocyte immunophenotype was observed in sirolimus-treated patients [43,47]. Interestingly, sirolimus was able to induce increased expression of the autophagy-associated ULK1 gene [20]. Altogether, these studies support the concept that sirolimus should be considered a double-acting molecule, inducing increased expression of the γ-globin gene (and associated increased production of fetal hemoglobin) on one hand and, on the other hand, increased expression of genes associated with autophagy activation and reduction of the excess of *α*-globin chains.

In conclusion, the data obtained indicated that low doses of sirolimus modify hematopoiesis and induce increased expression of γ-globin genes in a subset of patients with β-thalassemia. The analysis of molecular mechanism(s) able to limit the toxic effects of the excess of free *α*-globin chains was not analyzed by Zurlo et al., focusing their experimental effort only on *ULK1* gene expression. In order to expand the research on this very important field of investigation, the content of *AHSP* mRNA was determined in the present study by RT-qPCR in ErPCs isolated from sirolimus-treated patients participating to the Sirthalaclin clinical trial.

### 3.4. Sirolimus (Rapamycin) Treatment of β-Thalassemia Patients Participating to the Sirthalaclin NCT03877809 Trial Induces an Increase in AHSP mRNA Production in ErPCs from Sirolimus-Treated β-Thalassemia Patients

**Figure 4:**
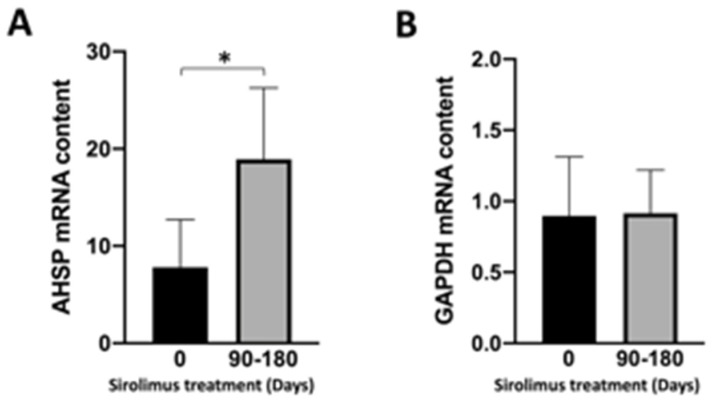
*AHSP* RNA content in ErPCs isolated from sirolimus-treated β-thalassemia patients participating to the Sirthalaclin (NCT03877809) clinical trial. The genotypes of the recruited patients have already been reported by Zuccato et al. [45]. The quantification of *AHSP* mRNA was performed using β-actin as internal reference sequence, as elsewhere described. * = *p* < 0.05 (statistically significant). Data and relative analyses were generated using the Prism Software v9.02 and paired *t*-test.

Figure 4A indicates that *AHSP* mRNA content was found to be increased in ErPCs from sirolimus-treated β-thalassemia patients. By contrast, no major changes in the content of *GAPDH* mRNA were detected (Figure 4B). These data were reproducible and support the concept that after 90–180 days of sirolimus treatment, *AHSP* RNA content increased with respect to ErPCs isolated from the same patients before starting sirolimus treatment.

## 4. Discussion

The α-hemoglobin stabilizing protein (AHSP) is an abundant erythroid chaperone that stabilizes free α-globin [22]. The level of expression of AHSP has a recognized impact on the severity of β-thalassemia [23,24,25,26,27,28,29]. In this respect, Kong et al. generated *AHSP*^–/–^ mice by gene targeting and reported that loss of AHSP impairs erythropoiesis and exacerbates β-thalassemia [24]. This was demonstrated by studying *AHSP*^−/−^ erythrocytes, which contained hemoglobin precipitates and exhibited cytotoxicity and decreased lifespan. In hematopoietic tissues, a high number of erythroid precursors exhibited activation of the apoptotic process. Consistent with unstable alpha-Hb, *AHSP*^−/−^, erythrocytes contained increased ROS and evidence of oxidative damage [24].

Conversely, high expression of AHSP was found to be associated with milder forms of β-thalassemia. In a study performed on patients with β thalassemia and sickle cell anemia, Mahmoud et al. [29] found significantly higher levels of AHSP in non-transfusion-dependent patients with β thalassemia (NTDT) compared to transfusion-dependent ones. Considering the importance of AHSP for the lifespan of erythroid cells, AHSP inducers are expected to be of interest from the therapeutic point of view. Concerning this issue, AHSP was found to be expressed at higher levels in β-thalassemia patients on hydroxyurea therapy [29]. In addition, Nitidine Chloride (NC) was found by Liu et al. [49] able to induce erythroid differentiation of human leukemic K562 cells, together with increased expression of erythroid differentiation markers, including AHSP. It should be interesting to verify whether NC can induce increased expression of AHSP in erythroid precursor cells from patients with β-thalassemia, especially those exhibiting excess of free α-globin chains. In a more recent study, Han et al. demonstrated that AHSP expression in K562 cells could be stimulated by Nrf2 (NFE2-related factor 2) and its agonist tert-butylhydroquinone (tBHQ) [50]. In this context, sulforaphane (SFN) is a natural activator of the Nrf2/Keap1 cytoprotective pathway [51]. Other NRF2 activators have been reported and recently reviewed [52].

These are the major conclusion of our study: RT-qPCR and Western blotting experiments demonstrate that *AHSP* gene expression is higher in erythroid precursor cells (ErPCs) isolated from β-thalassemia patients (Figure 1), and that its expression, analyzed by RT-qPCR, is enhanced by treatment with sirolimus (Figure 2). We had the possibility to verify the effects of sirolimus on *AHSP* gene expression in vivo by studying the accumulation of *AHSP* mRNA in ErPCs isolated from sirolimus-treated β-thalassemia patients participating in the Sirthalaclin (NCT03877809) clinical trial. The RT-qPCR data obtained are shown in Figure 4 and indicate a statistically significant (*p* < 0.05) increase in *AHSP* mRNA when ErPCs from sirolimus-treated patients are compared to ErPCs taken from patients before the beginning of the daily intake of sirolimus.

To our knowledge, this is the first report demonstrating the ability of sirolimus to stimulate AHSP expression ex vivo (Figure 2) and in vivo (Figure 4).

Our study indicates that sirolimus (rapamycin) should be considered among the AHSP inducers, both in vitro and in vivo. Therefore, sirolimus should be considered a double-acting drug, able to induce HbF and, at the same time, pathways responsible for reversing the toxic effects of the excess production of free α-globin chains, such as the autophagy [11,12] and AHSP pathways.

**Figure 5:**
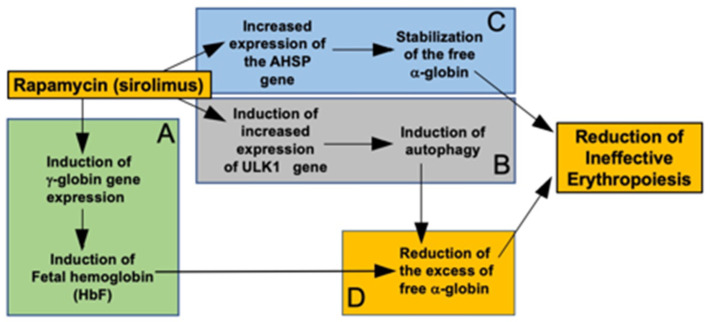
Pictorial representation of the proposed mechanism of action of sirolimus (rapamycin). Sirolimus induces increased expression of γ-globin genes and increased production of HbF (**A**) [35,36,43]. In addition, sirolimus induced increased expression of the *ULK1* gene, leading to induction of autophagy [19,20] (**B**) and increased expression of the *AHSP* gene, leading to stabilization of the free α-globin (**C**) [Figure 2 and Figure 4] (**C**). Induction of fetal hemoglobin and autophagy cooperate in reducing the excess of free α-globin (**D**). AHSP-dependent stabilization of the free α-globin, and ULK1/autophagy-dependent reduction of the excess of free α-globin might concur in the reduction of ineffective erythropoiesis.

We would like to underline that rapamycin is also able to enhance the expression of the *ULK1* gene in erythroid precursors from β-thalassemia patients. The proposed mechanism of action of sirolimus (rapamycin) is presented in Figure 5.

### 4.1. Strength of the Study

This study’s strength is that, to the best of our knowledge, this is the first report showing induction of *AHSP* gene expression by sirolimus in β-thalassemic erythroid precursor cells. This was demonstrated in both ex vivo and in vivo experiments, taking advantage, in the case of the in vivo approach, of our research activity related to the Sirthalaclin clinical trial (NCT03877809).

### 4.2. Limitations and Drawbacks of the Study

This study has some important limitations and drawbacks. First of all, only sirolimus (rapamycin) was used as an HbF inducer. In this respect, in the near future, it would be very interesting to verify if other HbF inducers (for instance, hydroxyurea, mithramycin, resveratrol, azacytidine, decitabine, and others) [6,53] are able, as rapamycin, to induce an increase in *AHSP* gene expression in treated erythroid cells isolated from β-thalassemia patients.

A second but very important limitation of this study is the low number of patients recruited for obtaining ErPCs to be used. Therefore, we were unable to draw any conclusions about the relationship between genotype and AHSP gene expression. Furthermore, regarding the samples obtained from the patient population of the Sirthalaclin clinical trial (NCT03877809)), they complied with the inclusion criteria of the trial [42,43].

### 4.3. Future Research Efforts and Perspectives

With respect to the above-mentioned limitations and drawbacks, more extensive studies based on ErPCs isolated from much larger cohorts of β-thalassemia patients will allow to create study groups, homogenous for genotype, basal levels of HbF production, blood transfusion levels, and HbF-associated polymorphisms (for instance the *XmnI,* the *BCL11A* and *HBS1L-MYB* single nucleotide polymorphisms, known to be associated to response to HbF inducers in pharmacogenomic studies). This will allow, on one hand, clarifying how general is the rapamycin-associated increase in *AHSP* gene expression in treated β-thalassemia erythroid cells; on the other hand, this will allow studying possible relationship(s) between *AHSP* gene expression and key genetic and phenotypic features of β-thalassemia, including the extent of increased expression of γ-globin genes.

Finally, a very interesting issue will be to verify whether the ULK1 (Figure 5B) and the AHSP (Figure 5C) network can be co-activated in erythroid cells from β-thalassemia patients. This very interesting possibility will open a new field of research activities focusing on the possible interplay between *AHSP* and *ULK1* gene expression.

## 5. Conclusions

Our study suggests that sirolimus (rapamycin) might induce increased production of α-hemoglobin stabilizing protein (AHSP) in erythroid precursor cells (ErPCs) from β-thalassemia patients. Two approaches were considered to verify sirolimus (rapamycin) effects on the expression of AHSP in ErPCs. The first was an ex vivo approach. ErPCs were isolated from β-thalassemia patients and then treated with a low concentration of rapamycin (200 nM). The second was an in vivo approach, in which the analysis was conducted on ErPCs isolated from sirolimus-treated β-thalassemia patients participating to the Sirthalaclin clinical trial (NCT03877809). Although our exploratory study has been conducted on a relatively low number of patients, the results obtained are concurrently suggestive of increased expression of the *AHSP* gene in ErPCs after treatment with sirolimus, both ex vivo and in vivo. Therefore, we propose to extend the study on *AHSP* gene expression in rapamycin-treated ErPCs in order to render our observations more representative; if the observation presented in this study is confirmed, we suggest that AHSP gene expression should be considered in future clinical trials based on fetal hemoglobin inducers.

## Figures and Tables

**Table 1:** List of primers and probes with related sequences used to perform RT-qPCR analyses.

Primers and Probes	Sequences
primer forward *AHSP*	5′-GAGACATATACAGCCTGTTAGACC-3′
primer reverse *AHSP*	5′-GAGGATCATTGAAGACCTGCT-3′
probe *AHSP*	5′-FAM-ATGAGATCCTTATTGGCCTTAAGAAGAGCC-BFQ-3′
primer forward *RPL13A*	5′-GGCAATTTCTACAGAAACAAGTTG-3′
primer reverse *RPL13A*	5′-GTTTTGTGGGGCAGCATACC-3′
probe *RPL13A*	5′-HEX-CGCACGGTCCGCCAGAAGAT-BFQ-3′
primer forward β*-actin*	5′-ACAGAGCCTCGCCTTTG-3′
primer reverse β*-actin*	5′-ACGATGGAGGGGAAGACG-3′
probe β*-actin*	5′-Cy5-CCTTGCACATGCCGGAGCC-BRQ-3′
primer forward *GAPDH*	5′-ACATCGCTCAGACACCATG-3′
primer reverse *GAPDH*	5′-TGTAGTTGAGGTCAATGAAGGG-3′
probe *GAPDH*	5′-FAM-AAGGTCGGAGTCAACGGATTTGGTC-BFQ-3′

**Table 2:** Western blot primary and secondary antibodies employed for detection of protein present in ErPCs lysates.

Target	Primary Antibody	Cat.n.	Secondary Antibody	Cat.n.
AHSP	Rabbit anti-AHSP (ABclonal, Woburn, MA, USA)	A6465	Mouse Anti-rabbit IgG HRP (Cell Signalling Technology, Danvers, MA, USA	7074
GAPDH	Mouse anti-GAPDH (Thermo Fisher, Waltham, MA, USA)	MA1-16783	Goat Anti-mouse IgG HRP (Thermo Fisher, Waltham, MA, USA)	32430

## Data Availability

Available materials and further information on the data will be freely available upon request to the corresponding authors.

## Supplementary Materials

The following supporting information can be downloaded at: https://www.mdpi.com/article/10.3390/jcm13092479/s1, Figure S1. Expression of AHSP in Erythroid Precursor Cells (ErPCs) isolated from healthy subjects and β-thalassemia patients. We report the RT-qPCR analysis shown in Figure 1 of the main text but normalized on RPL13A (A) and β-actin (B) as reference sequences, showing high reproducible data. Statistical data were generated using the Prism Software v9.02 and unpaired *t*-test. Figure S2. The uncropped version of Western Blot presented in Figure 1. In panel A we show the acquired blot image, in panel B the nitrocellulose membrane with the prestained multicolor protein ladder (Spectra pre-stained ladder by Thermo Fisher, Waltham, MA, USA, cat. n. 26634) and in panel C the merge of picture A and B, showing the exact molecular weight of the target proteins.
