# Supplementary material for: Increased Expression of α-Hemoglobin Stabilizing Protein (AHSP) mRNA in Erythroid Precursor Cells Isolated from β-Thalassemia Patients Treated with Sirolimus (Rapamycin)
Source: J Clin Med. 2024 Apr 24;13(9):2479. doi: 10.3390/jcm13092479 (PMC11084795; doi:10.3390/jcm13092479)
Supplement: Supplementary file 1 [file jcm-13-02479-s001.zip › jcm-2766620-supplementary.pdf]

Journal of
Clinical Medicine

MDPI

Article

Increased Expression of α-Hemoglobin Stabilizing Protein (AHSP) mRNA in Erythroid Precursor Cells Isolated from β-Thalassemia Patients Treated with Sirolimus (Rapamycin)

Matteo Zurlo [1], Cristina Zuccato [1,2], Lucia Carmela Cosenza [1], Maria Rita Gamberini [2], Alessia Finotti [1,2,*] and Roberto Gambari [1,2,*]

1 Department of Life Sciences and Biotechnology, Ferrara University, 44121 Ferrara, Italy; matteo.zurlo@unife.it (M.Z.); cristina.zuccato@unife.it (C.Z.); luciacarmela.cosenza@unife.it (L.C.C.)
2 Center "Chiara Gemmo and Elio Zago" for the Research on Thalassemia, Department of Life Sciences and Biotechnology, Ferrara University, 44121 Ferrara, Italy; gamberinimariarita@gmail.com
* Correspondence: alessia.finotti@unife.it (A.F.); gam@unife.it (R.G.)

SUPPLEMENTARY MATERIAL

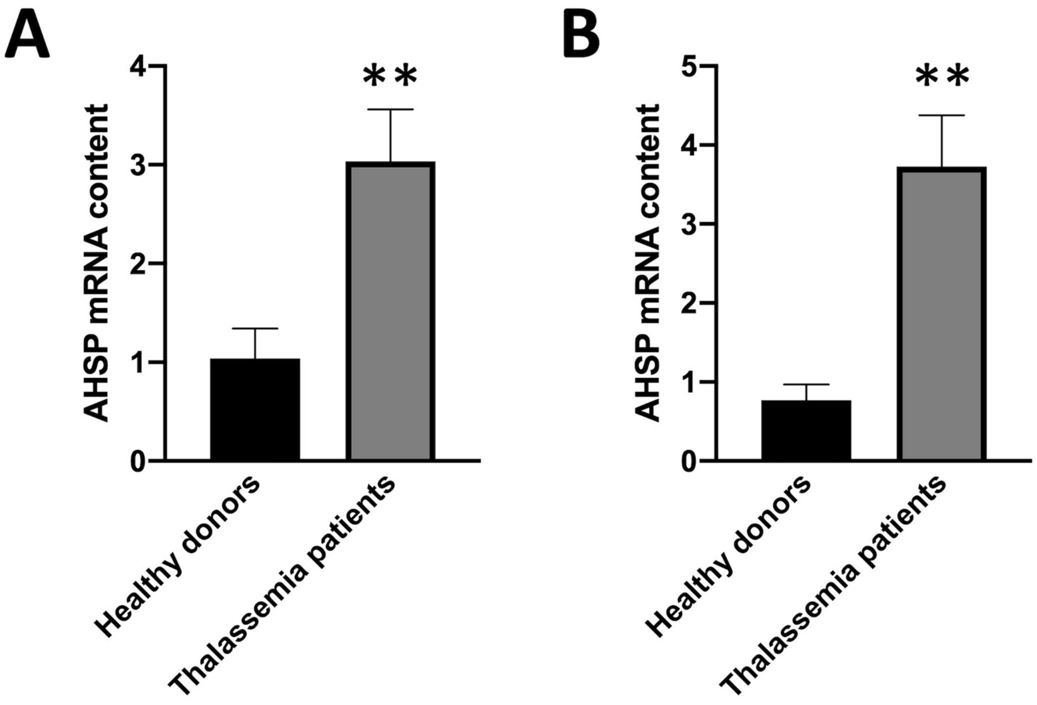

Figure S1. Expression of AHSP in Erythroid Precursor Cells (ErPCs) isolated from healthy subjects and β-thalassemia patients. We report the RT-qPCR analysis showed in Figure 1 of the main text but normalized on RPL13A (A) and β-actin (B) as reference sequences, showing high reproducible data. Statistical data were generated using the Prism Software v9.02 and unpaired t-test.

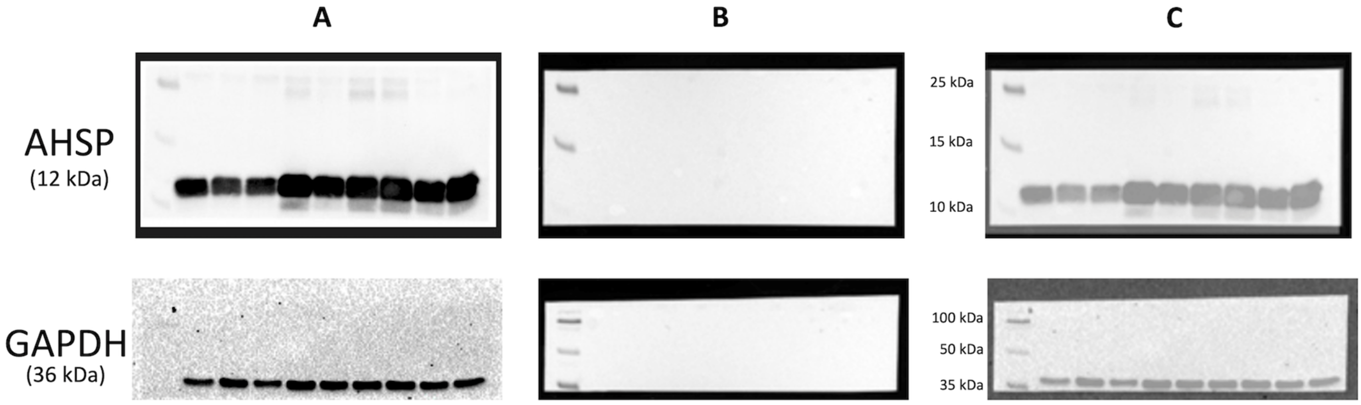

Figure S2. Uncropped version of Western Blot presented in Figure 1. In panel A we show the acquired blot image, in panel B the nitrocellulose membrane with the prestained multicolor protein ladder (Spectra pre-stained ladder by Thermo Fisher, Waltham, MA, USA, cat. n. 26634) and in panel C the merge of picture A and B, showing the exact molecular weight of the target proteins.
